# State Transfer via On-Line State Estimation and Lyapunov-Based Feedback Control for a *N*-Qubit System

**DOI:** 10.3390/e21080751

**Published:** 2019-07-31

**Authors:** Sajede Harraz, Shuang Cong

**Affiliations:** Department of Automation, University of Science and Technology of China, Hefei 230027, China

**Keywords:** quantum Lyapunov control, state transfer, quantum state estimation, quantum feedback control, on-line quantum state estimation

## Abstract

In this paper, we propose a Lyapunov-based state feedback control for state transfer based on the on-line quantum state estimation (OQSE). The OQSE is designed based on continuous weak measurements and compressed sensing. The controlled system is described by quantum master equation for open quantum systems, and the continuous measurement operators are derived according to the dynamic equation of system. The feedback control law is designed based on the Lyapunov stability theorem, and a strict proof of proposed control laws are given. At each sampling time, the state is estimated on-line, which is used to design the control law. The simulation experimental results show the effectiveness of the proposed feedback control strategy.

## 1. Introduction

Quantum control theory has attracted considerable attention both theoretically [1,2,3,4,5,6] and experimentally [7,8]. Most studies of quantum control are concerned with transferring the state of the system to a desired final state [9,10]. Quantum Lyapunov control (QLC) has been widely studied to control quantum systems for state preparation [11,12], trajectory tracking [13,14] and state transfer [15,16,17] with different Lyapunov functions [18,19]. In QLC, the control laws are designed by keeping the first-order time derivative of a selected Lyapunov function less than zero. The selection of the appropriate Lyapunov function is tricky, where different Lyapunov functions make different control effects. The best approach is to use some special geometric or physical meanings of the system to design the Lyapunov function. The most common Lyapunov functions are: Lyapunov function based on the state distance, on the average value of an imaginary mechanical quantity, and on the state error [18]. In [15,20] the control laws are designed using the distance-based Lyapunov function, and the conditions are obtained for the asymptotic stability of the closed-loop system by linearizing the unitary operator of the state. Using the QLC feedback control, one can achieve more accurate control performance. However, it should use the system’s state, which needs to be estimated on-line.

Quantum state estimation (QSE) is a useful tool which describes the characterization of the state of a quantum system [21]. To estimate the state of the system, one needs to apply appropriate measurements on the system and reconstruct the state based on the results of the measurements by an estimator algorithm. Different measurements and estimation methods have been well studied in the field of QSE. For instance, projective measurement [22,23], continuous weak measurement [24,25,26] and sequential unsharp measurement [27], which have been used with estimation approaches such as maximum likelihood or Bayesian method to reconstruct the initial state of the measurement processes [28,29]. These methods have been widely used in experiments [30,31]. Compressed quantum tomography, based on techniques from compressed sensing [23,32], reduces the required number of measurements to reconstruct the state. In classical control theory, one gets the best estimation of the state of the system at each time and uses the results of the estimation for controlling a closed loop system. The same feedback control is possible for quantum feedback control by using OQSE [33]. On-line quantum state estimation (OQSE) is a continuous state estimation at any moment by using continuous weak measurements with the help of some optimization algorithms. In OQSE, the results of continuous measurements are used to reconstruct the state of the system at any time. The measurement operators should be designed based on the dynamic equation of the system which changes over time. In [34], the authors proposed a quantum state estimation scheme and employed a continuous measurement protocol to perform QSE on the seven-dimensional, F=3 atomic hyperfine spin manifold, in an ensemble of cesium atoms. Then a state-to-state quantum mapping performance estimation based on the continuous weak measurement is achieved, as well as the optimal control technology design and implementation [35]. By using non-destructive measurements and the dynamics of the system, at the end of the estimation procedure, the state of the system was reconstructed. Hence, the result of OQSE can be used for real-time state feedback control. The dynamic of the open quantum system can be described using the Lindblad-type Markovian master equation [36]. In Markovian quantum control, any time delay is ignored and a memoryless controller is assumed. The measurement record is immediately fed back onto the system to alter the system dynamics [37].

In this paper, we propose a Lyapunov-based state feedback control based on the OQSE, to transfer the state of a *N*-qubit system to a desired final state for a Markovian open quantum system. The measurement operators for the *N*-qubit system are derived, which are indirectly acted on the controlled system and change as the time. The quantum state is estimated on-line based on the continuous weak measurements and compressed sensing, and the state feedback control law is designed based on the Lyapunov stability theorem, which is used to transfer the state of *N*-qubit Markovian system from an initial state to a desired final state. The control laws based on the Lyapunov stability theorem are designed with variable control parameters.

The layout of the paper is as follows. We derive the process of *N*-qubit OQSE in Section 2. Then, we design the Lyapunov-based feedback control based on on-line estimation in Section 3 and the numerical simulation is in Section 4. Finally, Section 5 presents the conclusions.

## 2. *N*-Qubit OQSE Establishment

In OQSE, the results of continuous weak measurements are used to reconstruct the state of the system in real time with compressed sensing theory. The measurement operators are derived by means of the weak measurement and the dynamic equation of the system. At each instant time, one can obtain the records of the expectation values with some measurement operators by the indirect results of continuous weak measurements. The estimated state can be obtained by solving convex optimization problem with physical constraints.

The *n*-dimensional dynamics of open quantum system can be described as

(1) $$\begin{array}{c} \rho \left(t+dt\right)-\rho \left(t\right)=-\frac{i}{\hbar }\left[H\left(t\right),\rho \left(t\right)\right]dt+\sum_{}\left[L\rho \left(t\right)L^{†}-\left(\frac{1}{2}L^{†}L\rho \left(t\right)+\frac{1}{2}\rho \left(t\right)L^{†}L\right)\right]dt\;,\;\\ \rho_{0}=\rho \left(0\right) \end{array}$$

where ρ(t) is the density matrix of the system which is a $2^{N}\times 2^{N}$ matrix, *N* the number of qubits and $n=2^{N}$ the dimension of the system; H(t) the total Hamiltonian of the system, and $H\left(t\right)\;=\;H_{0}\;+\;\sum_{j=1}^{m}U_{j}\left(t\right)H_{j}$; $H_{0}$ is the free Hamiltonian of the system, and $H_{j}$ is the control Hamiltonian. Let $D\left[L,\rho \right]=L\rho L^{†}-\left(1/2\right)\left(L^{†}L\rho +\rho L^{†}L\right)$, which is the decoherence effect of the measurement process, and manifests as the drift term of the Lindblad form.

For the continuous weak measurements of a two-level quantum systems, the measurement operator group contains two operators: $m_{0}\left(\Delta t\right)$ and $m_{1}\left(\Delta t\right)$ as [33]

(2) m0(Δt)=I−L†LL†L22+iH(t)Δtm1(Δt)=mk≠j=L·Δt

where Δt is the time for measurement and *L* is a Lindblad operator as

(3) L=ξB

where ξ is the strength of the measurement, and the operator *B* is chosen from the Stokes measurements set in this paper as:

(4) $$B_{0}=|H\rangle \langle H|+|V\rangle \langle V|,\;B_{1}=|H\rangle \langle H|,\;B_{2}=|D\rangle \langle D|,\;B_{3}=|R\rangle \langle R|$$

where $|H\rangle \equiv |0\rangle =\left(\begin{array}{c} 1\\ 0 \end{array}\right)$ is horizontal polarization, $|V\rangle \equiv |1\rangle =\left(\begin{array}{c} 0\\ 1 \end{array}\right)$ is vertical polarization, D≡H+VH+V22 is diagonal polarization, and R≡H+iVH+iV22 is right-circular polarization.

The discrete-time dynamic equation of the open quantum system is:

(5) $$\rho \left(t+1\right)=M_{0}\rho \left(t\right)M_{0}^{†}+M_{1}\rho \left(t\right)M_{1}^{†}+\ldots +M_{2^{N-1}}\rho \left(t\right)M_{2^{N-1}}^{†}$$

For *N*-qubit state estimation, one usually needs to use $2^{N}$ measurement operators, which are calculated by tensor products of $m_{0}$ and $m_{1}$ given in Equation (2):

(6) $$\begin{array}{c} M_{0}\left(\Delta t\right)=\underset{N}{\underset{︸}{m_{0}\left(\Delta t\right)⊗\ldots ⊗m_{0}\left(\Delta t\right)⊗m_{0}\left(\Delta t\right)}}\\ M_{1}\left(\Delta t\right)=\underset{N}{\underset{︸}{m_{0}\left(\Delta t\right)⊗\ldots ⊗m_{0}\left(\Delta t\right)⊗m_{1}\left(\Delta t\right)}}\\ \;\;\;\;\;\;\;\;\vdots \;\;\;\;\;\;\;\;\;\;\;\;\;\;\;\;\;\;\;\;\;\;\;\;\;\;\;\;\;\;\;\;\;\;\;\;\;\;\;\;\;\;\;\;\;\;\;\;\;\;\;\;\;\;\;\;\;\;\;\;\;\vdots \\ M_{2^{N}-1}\left(\Delta t\right)=\underset{N}{\underset{︸}{m_{1}\left(\Delta t\right)⊗\ldots ⊗m_{1}\left(\Delta t\right)⊗m_{1}\left(\Delta t\right)}} \end{array}$$

The corresponding discrete-time dynamic equation of continuous weak measurement operators for *N*-qubit is:

(7) $$M\left(t+1\right)=M_{0}{}^{†}M\left(t\right)M_{0}+M_{1}{}^{†}M\left(t\right)M_{1}+\ldots +M_{2^{N-1}}{}^{†}M\left(t\right)M_{2^{N-1}}$$

The reconstruction problem of density matrix ρ is transformed into the following optimization problem: $\min{\left\|\hat{\rho }\right\|}_{*}\;\;s.t.\;\;\;y=A.vec\left(\hat{\rho }\right)$, where ${\left\|\hat{\rho }\right\|}_{*}$ is the nuclear-norm of estimated density matrix $\hat{\rho }$, vec(·) represents the transformation from a matrix to a vector by stacking the matrix’s columns in order on the top of one another. The vector *y* and matrix *A* can be expressed according to the current measurement configurations as:

(8) $$y\left(l\right)={\left(\langle M(1)\;\rangle ,\langle M(2)\rangle ,\ldots ,\langle M(l)\rangle \right)}^{T},l=1,2,\ldots ,K$$

and

(9) $$A\left(l\right)=\left(\begin{array}{cccc} vec{\left(M\left(1\right)\right)}^{T} & vec{\left(M\left(2\right)\right)}^{T} & \cdots & vec{\left(M\left(l\right)\right)}^{T} \end{array}\right),l=1,2,\ldots ,K$$

where 〈M(l)〉, l=1,2,…,K is the corresponding measurement value in the *l*-th measurement; the sampling vector *y* is the vector form of the corresponding observation values 〈M(l)〉; and *K* is the selected number of measurements to estimate the state of the system accurately at each sampling time. The estimator needs to estimate density matrix parameters, which are $d^{2}$ elements of density matrix for a *d*-dimensional Hilbert space. Hence the maximum amount of *K* is $d^{2}$. To limit the number of measurements, we define the measurement rate as:

(10) $$\beta =\frac{K}{d^{2}}$$

where *K* is the number of measurements used in the state estimation and *d* is the dimension of Hilbert space. Hence, at each sampling time, we choose the last *K* number of measurements and discards the others. The measurement rate is proportional to the number of measurements and when $\beta =1(K=d^{2})$ the measurement set is an informationally complete set; and when the number of measurement outcomes is less than the number of elements $K<d^{2}$, the measurement set is called an informationally incomplete set.

To solve the optimization problem, we use the non-negative least squares estimator of minimizing the 2-norm under the positive definite constraint:

(11) $$\begin{array}{c} \arg\min{\left\|A\cdot vec(\hat{\rho })-y\right\|}_{2}\;\;\\ s.t.\;\;\;\hat{\rho }\geq 0\;,\;\mathrm{tr}(\hat{\rho })=1 \end{array}$$

To solve Equation (11) we used CVX, a package for specifying and solving convex programs [38,39].

In this paper, we use fidelity as the performance of OQSE which is defined as the trace between actual state and estimated state as:

(12) $$F=Tr\sqrt{\hat{\rho }{\left(t\right)}^{1/2}\rho \left(t\right)\hat{\rho }{\left(t\right)}^{1/2}}\;$$

where $\hat{\rho }\left(t\right)$ is the estimated state and ρ(t) the actual state of the system at sampling time *t*. Generally, fidelity $F\in \left[0,1\right]$ measures how much two states overlap each other. A fidelity of 1 means the states are identical, whereas the fidelity of 0 means the states are orthogonal.

## 3. Lyapunov-Based State Feedback Control

In this section, we propose the Lyapunov-based state feedback control to transfer the state from given initial state to the desired final state of Markovian open quantum systems. The control laws are designed based on the Lyapunov stability theorem and the theoretical proof is given.

**Theorem** **1.**  *For the dynamics equation of controlled system Equation (1), let* $T_{j}=tr\left(\left(i\left[H_{j},\hat{\rho }\left(t\right)\right]\right)\left(\hat{\rho }\left(t\right)-\rho_{f}\right)\right),\;\;j=1,2,\ldots ,m$ *where* $\hat{\rho }\left(t\right)$ *is the estimated state by OQSE,* $\rho_{f}$ *is the desired final state, m is the number of control fields which is* m≥2 *,* $C=tr\left(\left(D\left[L,\hat{\rho }\left(t\right)\right]-i\left[H_{0},\hat{\rho }\left(t\right)\right]\right)\left(\hat{\rho }\left(t\right)-\rho_{f}\right)\right)$ *and* θ=0.0005 *a small positive number.*

*The first control Hamiltonian is set equal to free Hamiltonian of the system* $H_{0}=H_{1}$ *, with control law* $U_{1}=-1$ *. The other control laws are designed as:*

*(1) If*$\left|T_{2}\right|>\theta$*, then the designed control law is*U2=−C−CT2T2to compensate*C*and*U*_*j*≠1,2_ = −*K_j_.T_j_* where *K_j_* is a positive tunable number that satisfies $\overset{\cdot }{V}=\sum_{j\neq 1,2}-K_{j}.T_{j}^{2}<0$. In this case, the control laws which can make the control system asymptotically stable are:

(13) U(t)=U2(t)⋮Uj(t)⋮Um(t)=−C−CT2T2⋮−Kj.Tj⋮−Km.Tm⋮

*(j) If* $\left|T_{2}\right|<\theta$ *, … and* $\left|T_{j}\right|>\theta$ *, we designed the control law* $U_{j}$ *bto counteract the drift C. Therefore, the control laws, which can make the control system asymptotically stable, become:*

(14) U(t)=U2(t)⋮Uj(t)⋮Um(t)=−K2.T2⋮−C−CTjTj⋮−Km.Tm⋮

*(m) If* $\left|T_{2}\right|<\theta$ *, …, and* $\left|T_{m}\right|>\theta$ *, we designed the control law* $U_{m}$ *to counteract the drift C. In this case, the control system becomes asymptotically stable by setting control laws as:*

(15) U(t)=U2(t)⋮Um(t)=−K2.T2⋮−C−CTmTm

*(m+1) If* $\left|T_{2}\right|<\theta$ *, …, and* $\left|T_{m}\right|<\theta$ *, the control laws which can make the control system asymptotically stable are:*

(16) $$U\left(t\right)=\left[\begin{array}{c} U_{2}\left(t\right)\\ \;\;\;\;\;\vdots \\ U_{m}\left(t\right) \end{array}\right]=\left[\begin{array}{c} g\\ \;\vdots \\ g \end{array}\right]$$

*where g is a small positive number.*

**Proof** **of** **Theorem** **1.**  Based on the Lyapunov stability theorem, one needs to select a scalar function V(X) with continuous partial derivatives which satisfies following conditions: (a) V(X) is a positive definite, V(x)≥0; (b) the first order time derivative of the Lyapunov function is negative: $\dot{V}\left(x\right)<0$. We select the trace distance as the Lyapunov function

(17) $$V\left(\hat{\rho }\left(t\right),t\right)=\frac{1}{2}tr\left({\left(\hat{\rho }\left(t\right)-\rho_{f}\left(t\right)\right)}^{2}\right)$$

Trace distance measures the closeness of two quantum states $\hat{\rho }$ and $\rho_{f}$. In the feedback control system, we need to make the amount of trace distance as small as possible to make sure that the system reaches the desired final state $\rho_{f}$.

The first time derivative of the Lyapunov function *V* can be calculated as:

(18) $$\begin{array}{c} \dot{V}\left(\hat{\rho }\left(t\right),t\right)=tr\left(\dot{\hat{\rho }}\left(t\right)\left(\hat{\rho }\left(t\right)-\rho_{f}\left(t\right)\right)\right)=\sum_{j=1}^{m}U_{j}\left(t\right)\;.\;tr\left(\left(i\left[H_{j},\hat{\rho }\left(t\right)\right]\right)\left(\hat{\rho }\left(t\right)-\rho_{f}\right)\right)\;\\ \;\;\;\;+tr\left(\left(D\left[L,\hat{\rho }\left(t\right)\right]-i\left[H_{0},\hat{\rho }\left(t\right)\right]\right)\left(\hat{\rho }\left(t\right)-\rho_{f}\right)\right)\\ \;\;\;\;=U_{1}\left(t\right)\;.\;T_{1}+\ldots +U_{j}\left(t\right)\;.\;T_{j}+\ldots +U_{m}\left(t\right)\;.\;T_{m}+C\\ \;\; \end{array}$$

where $T_{j}=tr\left(\left(i\left[H_{j},\hat{\rho }\left(t\right)\right]\right)\left(\hat{\rho }\left(t\right)-\rho_{f}\right)\right),\;\;j=1,2,\ldots ,m$ is a real function of the real time estimated state $\hat{\rho }\left(t\right)$ at time t; $C=tr\left(\left(D\left[L,\hat{\rho }\left(t\right)\right]-i\left[H_{0},\hat{\rho }\left(t\right)\right]\right)\left(\hat{\rho }\left(t\right)-\rho_{f}\right)\right)$ is a drift term which its sign cannot be determined.

By setting first control Hamiltonian equal to free Hamiltonian of the system $H_{0}=H_{1}$, with control law $U_{1}=-1$, the effects of free Hamiltonian of the system is compensated $H\left(t\right)=H_{0}-H_{1}+\;\ldots U_{j}\left(t\right)H_{j}+\;\ldots +U_{m}\left(t\right)H_{m}=U_{2}\left(t\right)H_{2}+\ldots +U_{m}\left(t\right)H_{m}$. Now one needs to design the control laws to transfer the state to the desired final state.

In each case of control law design, two crucial tasks should be done. (i) to compensate the influence of the drift term *C*. (ii) make sure that $\dot{V}\left(\hat{\rho }\left(t\right),t\right)<0$ holds. Hence, in each case of control law design, we compare $T_{j}$ with a small positive number θ=0.0005. If $T_{j}>\theta$ the control law $U_{j}$ choose to counteract the drift term *C*, as Uj(t)=−C−CTjTj; and other control laws are designed to hold the $\dot{V}\left(\hat{\rho }\left(t\right),t\right)<0$, as *U*_*j*_*(t)* = −*K_j_.T_j_* where *K_j_* is a positive tunable number that satisfies $\overset{\cdot }{V}=\sum_{j}-K_{j}.T_{j}^{2}<0$. By applying the designed control laws, the first time derivative of the Lyapunov function *V* in Equation (18) becomes:

(19) V˙(ρ^(t),t)=−C−CTqTqTq+C+∑j≠q−Kj.Tj.Tj=∑j≠q−Kj.Tj2<0

If none of the conditions in cases j=1,…,m satisfied, we need to apply a disturbance to the system. In case (m+1) where $\left|T_{2}\right|<\theta$, …, and $\left|T_{m}\right|<\theta$, means $T_{j}\approx 0$ and the system is in a stable point. In N-qubit system, there are two stable points $\rho_{st1}=diag{\left(\left[1,0\right]\right)}^{⊗N}$ and $\rho_{st2}=diag{\left(\left[0,1\right]\right)}^{⊗N}$. When the system is in one of these stable points $\hat{\rho }\left(t\right)=\rho_{st1}$ or $\hat{\rho }\left(t\right)=\rho_{st2}$, the first derivative of the Lyapunov function becomes always equal to zero $\dot{V}=tr\left(\dot{\hat{\rho }}\left(t\right)\left(\hat{\rho }\left(t\right)-\rho_{f}\left(t\right)\right)\right)=0$. In this case, we apply a disturbance to the system to bring it out of the stable point as given in Equation (16). □

The detailed control procedure is given in Algorithm 1.

**Algorithm 1** Pseudocode of state transfer based on OQSE and QLC**Require:** Initialize variables *L* in Equation (3), Hamiltonians *H*_0_, *H*_1_, *H*_2_ and Lyapunov control parameter *K_j_* in Equation (19).
1:**for**t= 1, 2, … **do**2: Set $H\left(t\right)=H_{0}+U_{1}\left(t\right)H_{1}+\ldots U_{j}\left(t\right)H_{j}+\ldots +U_{m}\left(t\right)H_{m}$.3: Calculate continuous measurement operators as in Equation (6).4: Find the state of the system ρ(t) as in Equation (5).5: Find the estimator variables y(l) and M(l) as in Equations (8) and (9).6: Find the estimated state $\rho_{e}$ by solving Equation (11) with CVX optimization.7: Find the performance of estimation (fidelity) as in Equation (12).8: Calculate the variables of Lyapunov function $T_{j},\;C$ as in (18).9: Set the control laws $U_{j}\left(t\right)$ as Equations (13)–(16).10: Find state distance *V* as in Equation (17).11:**endfor**


Steps 3 to 7 are related to OQSE and steps 8 to 10 are related to QLC. We note that at each sampling time, according to the designed control laws, a new Hamiltonian is defined (step 2 of the algorithm). Otherwise, the decoherence effect of the measurement process as defined in Equation (1), reduces the off-diagonal elements of the density matrix and leads the state to a maximally mixed state.

## 4. Numerical Simulations and Results Analyses

In this section, the experimental simulation results and analysis for N-qubit state transfer based on OQSE are given. In the simulation experiments we assume that each qubit is in the initial eigenstate $\rho_{in}=diag\left(\left[0,1\right]\right)$, and the desired final eigenstate is $\rho_{f}=diag\left(\left[1,0\right]\right)$. The initial and final states of N-qubit system are:

(20) $$\rho_{in}^{N}=\underset{N}{\underset{︸}{\rho_{in}⊗\cdots ⊗\rho_{in}}}\;\mathrm{and}\;\rho_{f}^{N}=\underset{N}{\underset{︸}{\rho_{f}⊗\cdots ⊗\rho_{f}}}\;$$

The free Hamiltonian and control Hamiltonians are defined as:

(21) $$H_{0}=\underset{N}{\underset{︸}{\sigma_{z}⊗\sigma_{z}⊗\cdots ⊗\sigma_{z}}}\;\;,\;H_{1}=\underset{N}{\underset{︸}{\sigma_{i}⊗\sigma_{i}⊗\cdots ⊗\sigma_{i}}}\;,\;\;H_{2}=\underset{N}{\underset{︸}{\sigma_{i}⊗\sigma_{i}⊗\cdots ⊗\sigma_{i}}}\;,\;\;\cdots ,H_{m}=\underset{N}{\underset{︸}{\sigma_{i}⊗\sigma_{i}⊗\cdots ⊗\sigma_{i}}}\;$$

where $\sigma_{i=1,2,3}$ are Pauli matrices as $\sigma_{x}=\left[\begin{array}{cc} 0 & 1\\ 1 & 0 \end{array}\right]$, $\sigma_{y}=\left[\begin{array}{cc} 0 & -i\\ i & 0 \end{array}\right]$ and $\sigma_{z}=\left[\begin{array}{cc} 1 & 0\\ 0 & -1 \end{array}\right]$.

To compensate for the effects of the free Hamiltonian of the system $H_{0}$, we design the first control Hamiltonian equal to free Hamiltonian of the system $H_{1}=H_{0}$ with control law $U_{1}=-1$. The other control Hamiltonians are set along *x* and *y* axis with designed Lyapunov control laws, given in Theorem 1 to transfer the state to the desired final state.

The weak measurement initial operator is set as:

(22) $$M\left(0\right)=\underset{N}{\underset{︸}{B_{i}⊗\cdots ⊗B_{i}}}\;\mathrm{and}\;L=\xi B_{i}$$

where $B_{i}$ are chosen from stokes measurement set given in Equation (4).

First, we do the experiment for a two-qubit system. The parameters for OQSE and Lyapunov control of two-qubit system are set as: the number of control fields is m=4, with the free Hamiltonian $H_{0}=\sigma_{z}⊗\sigma_{z}$ and the control Hamiltonians as $H_{1}=\sigma_{z}⊗\sigma_{z},\;\;H_{2}=\sigma_{y}⊗\sigma_{x},\;H_{3}=\sigma_{x}⊗\sigma_{y},H_{4}=\sigma_{x}⊗\left(\sigma_{x}+\sigma_{y}\right)\;$; the first control law is fixed as $u_{1}=-1$ at all sampling times to compensate the effects of free Hamiltonian of the system; the control law parameters are set as $K_{2}=16,K_{3}=25,K_{4}=30,\;g=0.25$; the measurement strength is set as ξ=0.7, the Lindblad operator as $L=\xi B_{3}$, and the initial weak measurement operator as $M\left(0\right)=B_{3}⊗B_{3}$. The measurement rate is set as β=0.25, which means at each sampling time, the last four measurements and corresponding results are used in the optimization algorithm.

**Figure 1:**
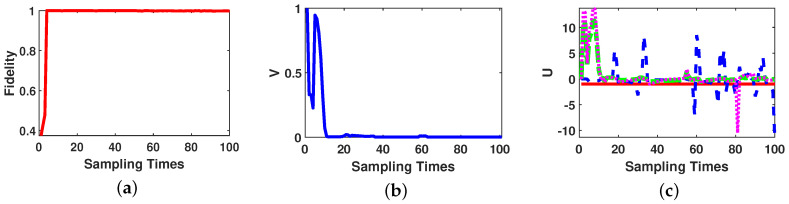
Two-qubit state transfer based on on-line estimation performance. (**a**) Fidelity between actual state and estimated state. (**b**) Trace distance between estimated state and the desired final state over the number of sampling times. (**c**) Variation curves of control law parameters. (The red solid line: $U_{1}$, the blue dashed line: $U_{2}$, the green dash-dotted line: $U_{3}$ and the pink dotted line: $U_{4}$).

Fidelity between actual state and estimated state is calculated according to Equation (12), and trace distance between estimated state and the desired final state as Equation (17). The behavior of fidelity, state distance and the control law parameters are given in Figure 1. We note that the fidelity evaluates the performance of the state estimation by comparing actual state and estimated state, and trace distance evaluates the performance of the transfer control by comparing the estimated state and the desired final state.

As Figure 1 shows, at sampling time 4, the amount of fidelity is close to 1, which means the OQSE estimates the state accurately; but the amount of trace distance is 0.22, which means the system could not reach the desired final state yet. After sampling time 11, the trace distance between the estimated state and the desired final state becomes less than 0.01; and after sampling time 40 the trace distance becomes less than 0.001. As Figure 1c depicted, the amount of first control law $U_{1}$ is always equal to −1 to compensate the effects of free Hamiltonian of the system and the other control laws are designed according to Lyapunov control method in Theorem 1.

**Figure 2:**
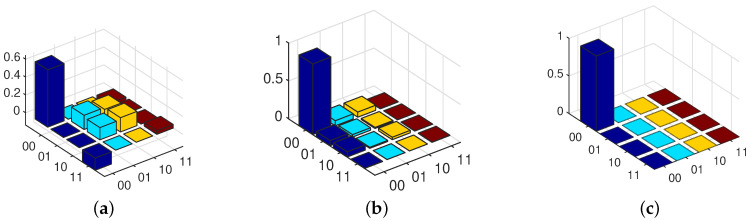
Two-qubit density matrix during sampling times. (**a**) Estimated state at sampling time 8. (**b**) Estimated state at sampling time 20. (**c**) Estimated state at sampling time 100.

To show the evolution of the state during the sampling times, Figure 2 shows the density matrix of the two-qubit system at 8, 20 and 100 sampling times.

As one can see from Figure 2, at sampling time 8, the state is far from the desired state (the trace distance is 0.61); however, as the sampling times increase, the state becomes closer to the desired final state. In a way, the density matrix at sampling time 20 becomes closer to the desired final state with trace distance 0.02; and at sampling time 100, the state is exactly same as the desired final state with trace distance 0.001.

**Figure 3:**
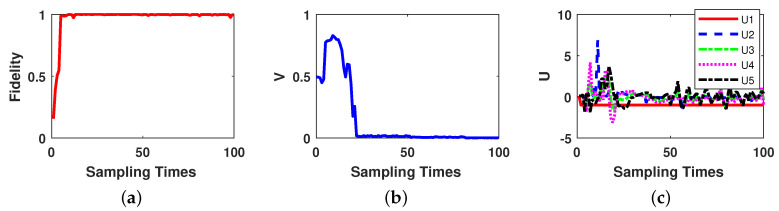
Three-qubit state transfer based on on-line estimation performance. (**a**) Fidelity between actual state and estimated state. (**b**) Trace distance between estimated state and the desired final state over the sampling times. (**c**) Variation curves of control law parameters.

Now we do the simulation experiment for a three-qubit system. The parameters for OQSE and Lyapunov control of three-qubit system are set as: the number of control fields m=5, where the free Hamiltonian is $H_{0}=\sigma_{z}⊗\sigma_{z}⊗\sigma_{z}$ and the control Hamiltonians are set as $H_{1}=\sigma_{z}⊗\sigma_{z}⊗\sigma_{z}$, which is same as free Hamiltonian with $U_{1}=-1$ to compensate the effects of free hamiltonian, $H_{2}\;=\;\sigma_{y}⊗\left(\sigma_{x}+\sigma_{y}\right)⊗\sigma_{y},\;H_{3}=\sigma_{x}⊗\sigma_{x}⊗\sigma_{y},H_{4}=\sigma_{x}⊗\sigma_{x}⊗\left(\sigma_{x}+\sigma_{y}\right)\;\mathrm{and}\;H_{5}=\left(\sigma_{x}+\sigma_{y}\right)⊗\sigma_{x}⊗\sigma_{y}\;$; the control law parameters are set as $K_{2}=25,K_{3}=28,K_{4}=22,K_{5}=30\;\mathrm{and}\;g=0.25$; the measurement strength is set as ξ=0.7, the Lindblad operator as $L=\xi B_{3}$, and the initial weak measurement operator as $M\left(0\right)=B_{3}⊗B_{3}⊗B_{3}$. The measurement rate is set as β=0.25, which means at each sampling time, the last 16 measurements and corresponding results are used in the optimization algorithm. The behavior of three-qubit state transfer control performance is given in Figure 3.

As Figure 3 depicts, the OQSE reaches the amount of fidelity more than 99% at sampling time 8; and the trace distance between estimated state and the desired final state becomes less than 0.01 at sampling time 24, and becomes less than 0.001 after sampling time 80. Compare with two-qubit system, for three-qubit system the feedback control needs longer sampling times to bring the state close to the desired final state. Two-qubit system trace distance is less than 0.01 after sampling time 11, but for three-qubit system, it happens after sampling time 24. Hence, we can conclude that for higher number of qubits, one can reach the desired final state in longer sampling times.

It is worth noting that the proposed state transfer control is applicable for any arbitrary initial and final states. However, one needs to find the suitable control parameters: control law parameters, Hamiltonians, measurement operators, measurement strength, Lindbald operator and initial measurement operator, for each initial and final states.

## 5. Conclusions

We designed the N-qubit state transfer control via OQSE and Lyapunov feedback control. The continuous weak measurement operators are designed based on the dynamic evolution of the system to estimate the state of the system in real time by compress sensing estimator. The control laws have been designed based on the Lyapunov stability theorem. The designed control laws can effectively achieve the state transfer of a N-qubit Markovian system from a given initial state to the desired target state. Fidelity between actual state and estimated state is defined to study the performance of the OQSE; and trace distance between estimated state and the desired final state is defined to study the performance of the state transfer control. The numerical simulation experiments show the effectiveness of the feedback control of N-qubit open quantum systems based on OQSE.
